# South African HIV self-testing policy and guidance considerations

**DOI:** 10.4102/sajhivmed.v18i1.775

**Published:** 2017-09-22

**Authors:** Francois Venter, Mohammed Majam, Lauren Jankelowitz, Siraaj Adams, Michelle Moorhouse, Sergio Carmona, Wendy Stevens, Busisiwe R. Msimanga, David Allen, Pooja Balani, Zwoitwaho Nevhutalu, Naleni Rhagnath, Amir Shroufi, Walter Devillé, Victoria Kazangarare, Renee van der Wiel, Hugo Templeman, Adrian Puren, Tim Tucker, Gilles van Cutsem, Francesca Conradie, Krista Dong, Thato Chidarikire, Andy Gray

**Affiliations:** 1 HIV Management Cluster, Wits Reproductive Health and HIV Institute, Johannesburg, South Africa; 2 Southern African HIV Clinicians Society, Johannesburg, South Africa; 3 Wits Reproductive Health and HIV Institute, University of the Witwatersrand, South Africa; 4 National Health Laboratory Service, Johannesburg, South Africa; 5 Department of Haematology, University of the Witwatersrand, South Africa; 6 World Health Organization, South Africa; 7 Bill and Melinda Gates Foundation, Johannesburg, South Africa; 8 South African National AIDS Council, Pretoria, South Africa; 9 Médecins Sans Frontières, Southern Africa; 10 Ndlovu Care Group, Groblersdal, South Africa; 11 Utrecht University Medical Center, Utrecht University, the Netherlands; 12 PSI, Johannesburg, South Africa; 13 Society for Family Health, Johannesburg, South Africa; 14 Wits Institute For Social & Economic Research, University of the Witwatersrand, South Africa; 15 National Institute for Communicable Diseases, Johannesburg, South Africa; 16 Strategic Evaluation, Advisory and Development Consulting (SEAD), Johannesburg, South Africa; 17 Department of Medicine, University of Cape Town, South Africa; 18 iTEACH, Denton, United States; 19 Massachusetts General Hospital, Boston, United States; 20 National Department of Health, Pretoria, South Africa; 21 Discipline of Pharmaceutical Sciences, University of KwaZulu-Natal, South Africa

## Abstract

The gap in HIV testing remains significant and new modalities such as HIV self-testing (HIVST) have been recommended to reach key and under-tested populations. In December 2016, the World Health Organization (WHO) released the Guidelines on HIV Self-Testing and Partner Notification: A Supplement to the Consolidated Guidelines on HIV Testing Services (HTS) and urged member countries to develop HIVST policy and regulatory frameworks. In South Africa, HIVST was included as a supplementary strategy in the National HIV Testing Services Policy in 2016, and recently, guidelines for HIVST were included in the South African National Strategic Plan for HIV, sexually transmitted infections and tuberculosis 2017–2022. This document serves as an additional guidance for the National HIV Testing Services Policy 2016, with specific focus on HIVST. It is intended for policy advocates, clinical and non-clinical HTS providers, health facility managers and healthcare providers in private and public health facilities, non-governmental, community-based and faith-based organisations involved in HTS and outreach, device manufacturers, workplace programmes and institutes of higher education.

## Introduction

### Background

HIV represents the primary burden of disease in South Africa, with an estimated national prevalence of 12.2% in 2012. The HIV annual incidence among individuals aged between 15 years and 49 years is estimated at 1.9%, and 2.3% among youth aged between 15 years and 24 years.

The country has a generalised and maturing HIV epidemic, with the highest number of people (6.4 million) living with HIV in the world. The prevalence of HIV in South Africa remains high. It is estimated that approximately 3 million people are on antiretroviral treatment (ART), making it the largest programme in the world. This can be attributed to the rapid scale-up and success of the ART programme. In South Africa, the proportion of people who have had an HIV test and are aware of their status has increased from 50% in 2008 to 66.5% in 2014. In the country there still remains a gap in HIV testing, and new approaches must be considered.

According to the World Health Organization (WHO), HIV self-testing (HIVST) is defined as the process whereby an individual collects their own specimen (blood or oral fluid), performs HIV testing using an HIV rapid diagnostic test (RDT) and interprets the result themselves either assisted or unassisted.^1^ HIVST does not provide a definitive diagnosis. A reactive (positive) result always requires a further confirmatory testing from a trained health professional using the relevant validated national HIV testing algorithm, typically performed at a clinic. Self-testing may also be termed ‘self-screening’.

In December 2016, the WHO released the Guidelines on HIV Self-Testing and Partner Notification: A Supplement to the Consolidated Guidelines on HIV Testing Services (HTS).^1^ These globally recognised guidelines have paved the way for in-country policy and guidance development on HIVST. In tandem, the WHO Diagnostics Prequalification Team released a Technical Specifications guidance document (TSS-1)^2^ for manufacturers who wish to apply for WHO Prequalification and diagnostic assessment of their HIVST product. Through this process, the WHO Prequalification sets the baseline for safety, quality and performance following international standards.

HIV self-testing has the potential to impact the first ‘90’ of the UNAIDS 90-90-90 targets, which have been adopted by South Africa, by increasing access and acceptability for traditionally under-tested populations. (UN 90-90-90 targets: by 2020, 90% of all people living with HIV will know their HIV status, 90% of all people with diagnosed HIV infection will receive sustained ART and 90% of all people on ART will have viral suppression.^3^). This includes key at-risk populations (serodiscordant couples, men, adolescents, female sex workers and men who have sex with men) who may require frequent or routine testing. HIVST is not intended to displace the confirmatory facility-based HIV test; rather HIVST is a complementary approach to existing HTSs, with the benefit of reaching previously untested, hard-to-reach and test-averse populations. HIVST has been shown to be widely acceptable among untrained users in various settings, particularly in key populations.^2,3^ To support the use of HIVST, guidelines for HIVST have recently been included in the South African National Strategic Plan for HIV, sexually transmitted infections (STIs) and tuberculosis (TB) 2017–2022.^4^ This comes after HIVST was included as a supplementary strategy in the National HIV Testing Services Policy for South Africa in 2016.

The majority of HIV kits for self-testing are based on HIV RDTs for professional use that have been modified and repackaged for an untrained layperson. Professional use HIV RDTs should not be used as an HIVST kit for the layperson without some level of modification, as there is a risk of misunderstanding or misinterpreting the instructions for use or test results. These tests should be validated for self-testing prior to use as such.

The ideal HIVST should be easier to use than the current HIV professional use tests. Self-test kits need to be made more ‘user-friendly’ and appropriate for an untrained, non-professional layperson. These modifications may include: revising the instructions for use to have fewer words and more pictures; simplifying specimen collection and transfer; reducing the number of process steps; achieving a faster time to results; ensuring that results remain stable for a longer period; making the test result easier to read and interpret; and optimising packaging and general instructions, including making this language-level and literacy-level appropriate. Each product must include referrals for assistance (telephone hotline, social media, local counsellor, phone application) with interpreting the results and linkage to care.

Innovations in information tools for referrals, such as online videos and apps, may improve test performance and linkage to confirmatory testing, prevention, treatment and care. With a few early HIV RDTs for self-testing already on the market more international HIVST products are emerging (HomeMed: http://www.homemed.co.za/hiv-1-or-2-single-test-kit/?p=Zz0xJmFw PTEmcD0x). However, substantial entry into the South African market has been inhibited by several barriers. Until recently,^5^ the most significant barrier has been the uncertainty around a national policy on HIVST, which has inhibited manufacturers from bringing high-quality products to the market. In the 2016 updated HIV counselling and testing guidelines, HIVST was recommended as a new approach for HIV testing (National HIV Testing Services Policy 2016).^6^ The HIVST agenda has moved rapidly and the evidence base to support HIVST has grown significantly (www.HIVST.org).

A glossary of terms used is provided in Appendix 1, and a list of acronyms and abbreviations in Appendix 2.

### Rationale for HIV self-testing guidelines

These guidelines serve as additional guidance for the National HIV Testing Services Policy 2016 with specific focus on HIVST.

There is a global initiative to accelerate universal access to HIV prevention, treatment and care. HIV testing remains the key point of entry in the continuum of care for any individual living with HIV. The gap in HIV testing remains significant and new modalities such as HIVST have been recommended as alternative approaches to traditional HTSs to reach key and under-tested populations.

The WHO’s formal recommendation states^1^: ‘HIV self-testing should be offered as an additional approach to HIV Testing Services (strong recommendation, moderate quality evidence)’.

In South Africa, HIV tests are available for purchase, through online channels, retail outlets and private community pharmacies. The quality of some of these tests is unknown. In February 2016, the South African Pharmacy Council lifted the ban on the sale of HIVST by pharmacists and has since published draft guidance on the provision of such tests by pharmacists.^7^ The Medicines Control Council, which will shortly become the South African Health Products Regulatory Authority (SAHPRA), has published regulations on medical devices and *in vitro* diagnostics.^8^ A risk-based approach will be followed in applying these new regulations, and HIV tests are expected to receive priority attention.

The WHO has urged member countries to develop *policy and regulatory frameworks* for HIVST.

These include adapting, developing and harmonising existing national policies on HIV testing to incorporate HIVST, such as:

- laws permitting the sale, distribution, advertisement and use of quality-assured RDTs for HIVST
- age of consent to self-test
- human rights laws, policies and regulations to protect individuals and address misuse of HIVST
- national policies on how to confirm an individual’s HIV status following HIVST
- quality assurance and post-market surveillance systems for RDTs used for HIVST.

### Target audience

This document is intended for policy advocates, clinical and non-clinical HTS providers, health facility managers and healthcare providers in private and public health facilities, non-governmental, community-based and faith-based organisations involved in HTS and outreach, device manufacturers, workplace programmes and institutes of higher education. The Department of Health, medical aids, non-governmental organisations, faith-based organisations and other organisations have to balance complex decisions around resource allocation, cost-effectiveness and expanded access to hard-to-reach populations.

### Guiding principles

All forms of HTSs, including HIVST, should adhere to the WHO 5Cs: Consent, Confidentiality, Counselling, Correct test results and Connection. These guiding principles are found in the National HIV Testing Services Policy 2016.^6^

## Key messaging from the WHO normative guidelines on HIV self-testing

**FIGURE 1:**
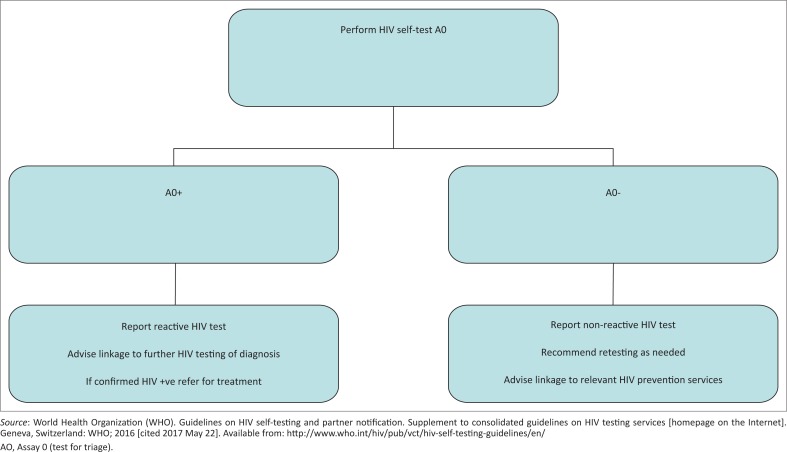
HIV self-testing algorithm.

The selected key points summarise the WHO’s HIVST guidelines^1^:

- HIVST is considered a Test for Triage (A0) (Figure 1) because a single RDT is not sufficient to make an HIV-positive diagnosis.
- A reactive (positive) self-test result always requires further testing and confirmation from a trained tester starting from the beginning of a validated national testing algorithm. Clear messages are essential to ensure users understand that HIVST does not provide a definitive HIV-positive diagnosis, and they are aware of what to do after a reactive self-test result.
- Interpretation of a non-reactive (negative) self-test result will depend on the ongoing risk of HIV exposure. Individuals at high ongoing risk, or who test within six weeks of possible HIV exposure, should be encouraged to re-test. HIVST is not recommended for users with a known HIV status who are taking antiretroviral drugs, as this may lead to an incorrect self-test result (false non-reactive).
- HIVST is acceptable to many users across different contexts and can, therefore, increase uptake and frequency of HIV testing, particularly among populations at high ongoing risk of HIV, who may be less likely to access testing or test less frequently than recommended.
- HIV RDTs used by self-testers can perform as accurately as when used by a trained tester, provided the HIVST products meet quality, safety and performance standards. In-person demonstrations and other support tools, such as videos, may also enhance the performance of HIVST.
- HIVST can be delivered through various approaches in the public and private sectors, including community-based, facility-based and internet-based channels. Approaches may also offer the option of using an oral-fluid-based or blood-based HIV RDT for self-testing. As such, different populations can benefit from a range of choices when self-testing for HIV.

## HIV self-testing rapid diagnostic tests

Evidence has shown that HIVST has a high level of test accuracy, in terms of both sensitivity and specificity, when quality-assured products are used in the hands of untrained users. The technology landscape is driving towards ensuring that HIVST products are fit for purpose and can demonstrate high accuracy when compared to use by a trained user.

### Target product profile

Adapted from the Global Target Product Profile (TPP)^9^ is a set of characteristics that the ‘ideal’ HIVST should exhibit. Device manufacturers must strive towards achieving and demonstrating the following:

- high-quality manufacturing standards, according to ISO 9001
- acceptable analytical performance in laboratory settings
- high clinical sensitivity and specificity in the hands of untrained users
- pictorial instructions for use with any text-based instruction translated into local languages
- low number of test steps with simple sample transfer
- simple-to-interpret test results
- fast time to result
- stable test results.

### Technology landscape

There are now several countries in which HIVSTs are available in the open market. These include USA, UK, Brazil, Australia and France. The products that are available for sale and distribution in these countries have been approved by a member of the Global Harmonization Task Force such as the FDA in the USA, and CE Mark in Europe. The FDA is widely considered to have the most stringent regulatory framework globally. Each member regulatory body sets out individual standards for product performance that must be met.

In South Africa, until the new regulatory framework for medical devices and *in vitro* diagnostics is fully operative and has addressed HIVSTs, the National Department of Health (NDoH) recommends that only HIVSTs that have been approved for the purposes of HIVST by the WHO Prequalification programme should be procured or sold. Under the requirements of the programme, manufacturers have to demonstrate high analytical performance, usability and clinical performance in the hands of untrained users. The full list of HIVSTs in the pipeline and those that have been approved can be viewed at: http://unitaid.eu/assets/HIV_rapid_diagnostic_tests_for_self-testing_-_semi-annual_update-december_2016.pdf.

### Guidance for HIV self-testing in South Africa

Based on the international guidelines above, a committee was convened on 17 November 2016 to establish guidelines appropriate for South Africa. Representatives at this meeting are included in Table 1-A3 available in Appendix 3.

The proposed guidelines for HIVST follow, towards an overarching goal of ensuring that high-quality, safe and effective products are available in the market:

- South Africa, through the Health Products Regulatory Authority, will develop a regulatory framework for Medical Devices, which will include HIVSTs. Until the development of this framework, HIVSTs must comply with the requirements of this guidance document.
- Products should strive to meet all of the requirements of the Target Product Profile, as per section ‘Target product profile’ above.
- Stable test results within the specified read window.
- The need for appropriate, validated, clear and concise instructions for use. This is critical in terms of a product’s usability to ensure that critical errors are minimised and accuracy is maximised. Manufacturers must demonstrate reasonable usability of their products in the region where they are marketed.
- Instructions for use and packaging materials must be translated and available in local languages, as well as in English.
- Clear messaging: Users must understand that a reactive or positive result must be confirmed through further testing by a healthcare worker, as well as where to access services.
- Manufacturers should include a ‘care card’ with the packaging that the user can take to a local clinic as evidence of having self-tested. Care card information should include the NDoH logo, the contact details for the National AIDS Helpline, the HIVST website (www.hivselftesting.co.za) or the manufacturer’s website that can provide any additional information on linkage to care as needed.
- Verification and certification of medical devices or *in vitro* diagnostic devices (IVDs) before they are placed on the market by manufacturers – all products must be accredited on the basis of a conformity assessment certificate issued by a Conformity Assessment Body, a body corporate or other legal entity, locally or internationally, accredited either by the South African National Accreditation System (SANAS) or an international body recognised by the Medicines Control Council, according to a standard determined by the Council, as competent to carry out the assessment.
- Post-marketing surveillance and lot number tracking to ensure device failures can be reported, lot numbers may be recalled and manufacturing quality can be assured. All kits must clearly show the expiry date of the product.
- All HIVST devices must provide disposal instructions and disposal supplies, e.g. sealable plastic bags.

## Implementation

### Implementation considerations

To maximise the benefit of HIVST, it is important not only to consider the products used but also how this modality may be implemented or scaled up. There are several mechanisms through which the service may be delivered, but there are also key considerations for all stakeholders such as linkage to care, quality control and monitoring and evaluation (M&E).

There are several models through which HIVST may be delivered and will depend on the target population:

These would include primary healthcare facilities, hospitals and pharmacies. Less traditional access points could be through internet distribution, workplace programmes and vending machines. In terms of community-based outreach, self-testing can be offered as part of a package to clients visiting pre-exposure prophylaxis facilities, centres for voluntary medical male circumcision, sexual and reproductive health, outreach clinics for STIs and TB and multi-disease prevention campaigns.

Secondary distribution includes peer-to-peer, and couples and partners.

Within the spectrum of channels for distribution of HIVST, there are also varying levels and types of support that can be offered to self-testers. These include: brief demonstrations on kit usage and result interpretation, face-to-face assistance during initial testing, and instructions for use. The following tools can be utilised for assistance or demonstration:

- Pictorials
- Videos
- Hotline
- Apps
- Multimedia
- SMS.

To facilitate linkage into care for individuals who receive a reactive self-test result, a care card bearing the NDoH logo should be implemented and standardised across the country, and should read (suggested wording):

Dear Sister in-charge,

This participant has screened for HIV using an HIV self-test and may have received a reactive (positive) result. Please ensure that the individual is provided with confirmatory testing and counselling should they require. (TWG)

### Monitoring and evaluation

HIV self-testing provides some unique challenges in terms of M&E, and globally there are not many systems outside of research-controlled environments that can effectively provide this. This is an area that the technical working group of the WHO is actively pursuing. The mere notion of a self-test shifts the focus away from a healthcare facility to an individual. Measurement of uptake cannot be inextricably linked to usage, as individuals who receive a test may not necessarily use it. The following have been proposed as minimum M&E during the initial roll-out of HIVST:

- periodical assessment of the hotline for HIVST-related calls requesting information, assistance, counselling and support
- reporting of adverse events to the national hotline, on dedicated web and social media platforms
- post-marketing surveillance data of companies
- linkage to care through e-health platforms.

### Implementation messaging

Successful implementation of HIVST requires clear messaging for both healthcare providers and end-users. The following messaging should be considered during implementation:

- An HIVST is a screening test. An initially reactive or positive self-test result requires further testing and confirmation from a trained tester. Clear messages are essential to ensure users understand that HIVST does not provide a definitive HIV-positive diagnosis, and they are aware of what to do after a reactive self-test result.
- A non-reactive or negative self-test result does not always indicate that a person is HIV-negative, depending on the test used, window period and also possible errors in performing the self-test or interpreting results, and, as with all HIV testing, individuals who have had a high-risk encounter in the preceding three months should be advised to re-test. Individuals at high, ongoing risk should be advised to re-test frequently.
- If an individual is on antiretroviral drugs for treatment or pre-exposure prophylaxis, this may lead to a false non-reactive or negative result, particularly if using an oral fluid-based RDT.
- To minimise social harm, test kits or care cards or packaging must contain the following statements:
  ■ This test is private, confidential and voluntary. If you are concerned about the outcome of the result or uncomfortable using the test DO NOT USE IT. Go to a clinic for testing.
  ■ If you require assistance telling your partner your result, then call the helpline or go to your nearest clinic.
  ■ Warning about disinhibition if HIV-negative and risk.
- Emphasis must be placed on the correct disposal of used HIVSTs.

## Appendix 1

### Glossary of terms

**Acute infection:** the period in which an individual becomes HIV-infected and before HIV antibodies can be detected by a serological assay.

**Assay:** a complete procedure for detecting the presence or concentration of an analyte, including all the components of a test kit used to identify HIV p24 antigen or HIV-1/2 antibodies, in the case of HIV.

**Confirm:** to issue a report on HIV status. Initially reactive test results, including reactive self-test results, need to be confirmed according to the national validated testing algorithm.

**Directly assisted HIV self-testing (HIVST):** refers to when individuals who are self-testing for HIV receive an in-person demonstration from a trained provider or peer before or during HIVST, with instructions on how to perform a self-test and how to interpret the self-test result. This assistance is provided in addition to the manufacturer-supplied instructions for use and other materials found inside HIVST kits.

**Harm or social harm:** any intended or unintended cause of physical, economic, emotional or psychosocial injury or hurt from one person to another, a person to themselves, or an institution to a person, occurring before, during or after testing for HIV.

**HIV self-testing:** a process in which a person collects his or her own specimen (oral fluid or blood) and then performs a test and interprets the result, often in a private setting, either alone or with someone he or she trusts.

**HIV status:** is the final report that is given to the patient; it is the final interpretation of the patient disease state and is based on a collection of testing results generated from one or more assays. HIV status may be reported as HIV-positive, HIV-negative or HIV-inconclusive.

**HIV test result:** the result from a single test on a given assay.

***In vitro* diagnostic medical device:** a medical device, used alone or in combination, intended by the manufacturer for the examination of specimens derived from the human body solely, or principally, to provide information for diagnosis, monitoring or determining compatibility. For example, an *in vitro* diagnostic medical device can be used for: diagnosis, as an aid to diagnosis, screening, monitoring, predisposition, prognosis, prediction and determination of physiological status.

**Key populations:** defined groups who, because of specific higher-risk behaviours, are at increased risk of HIV irrespective of the epidemic type or local context. These guidelines refer to the following groups as key populations: men who have sex with men, people who inject drugs, people in prisons and other closed settings, sex workers and transgender people.

**Non-reactive test result:** a test result that does not show a reaction indicating the presence of analyte, which in the context of HIV refers to HIV-1 p24 antigen or HIV-1/2 antibodies.

**Point-of-sex testing:** refers to when individuals use an HIV RDT for self-testing to screen potential sex partners and determine his or her own HIV status and their partner(s)’ HIV status.

**Quality assurance:** part of quality management focused on providing confidence among stakeholders that quality requirements will be fulfilled.

**Quality control:** is the set of procedures designed to monitor the test method and results to ensure appropriate test system performance. It includes testing control materials, charting the results and analysing them to identify source of error, and evaluating and documenting any remedial action taken as a result of this analysis.

**Quality improvement:** an element of quality management focused on increasing the ability to fulfil quality requirements.

**Quality management system:** a system to direct and control an organisation with regard to quality. Systematic and process-oriented efforts are essential to meet quality objectives. Principles of quality management include categories such as documents and records, organisation, personnel, equipment, purchasing and inventory, process control, information management, occurrence management, assessments (external and internal), process improvement, customer services and facilities and safety.

**Rapid diagnostic test:** *in vitro* diagnostic medical device of immunochromatographic or immunofiltration format for the detection of HIV-1/2 antibodies or HIV p24-1 antigen in the context of HIV.

**Reactive test result:** a test result that shows a reaction indicating the presence of analyte, which in the context of HIV includes HIV-1 p24 antigen or HIV-1/2 antibodies.

**Repeat testing:** a situation in which additional testing is performed for an individual immediately following a first test, during the same testing visit, because of HIV-inconclusive status or discordant test results. The same assay(s) is used and, where possible, the same specimen.

**Re-testing:** refers to certain situations in which individuals should be re-tested after a defined period of time: (1) HIV-negative people with recent or ongoing risk of exposure; (2) people with an HIV-inconclusive status; and (3) HIV-positive people before they enrol in care or initiate treatment. Reasons for re-testing before initiation of care or treatment include: ruling out laboratory or transcription errors, and ruling in or ruling out seroconversion.

**Sensitivity:** denotes the probability that an HIV assay or algorithm will correctly identify all specimens that contain HIV-1/2 antibodies or HIV-1 p24 antigen.

**Seroconversion:** is when an individual’s immune system produces a quantity of HIV-1/2 antibodies sufficient to be detectable on a given HIV serology assay.

**Serodiscordant couple:** a couple in which one partner is HIV-positive and one partner is HIV-negative.

**Serology assay:** refers to an assay that detects the presence of antibodies in human specimens. Such assays typically use serum or plasma, but also capillary or venous whole blood and oral fluid. For example, RDTs, immunoassays and certain supplemental HIV assays are serology assays.

**Specificity:** denotes the probability that the assay or algorithm will correctly identify specimens that do not contain HIV-1/2 antibodies or HIV-1 p24 antigen.

**Testing algorithm:** the combination and sequence of specific assays used within HIV testing strategies.

**Unassisted HIV self-testing:** refers to when individuals self-test for HIV using only a self-test kit that includes manufacturer-provided instructions for use. As with all self-testing, users may be provided with links or contact details to access additional support, such as telephone hotlines or instructional videos.

**Window period:** the period between HIV infection and the detection of HIV-1/2 antibodies using serology assays, which marks the end of the diagnostic window period and the end of seroconversion.

## Appendix 2

AIDS, Acquired immune deficiency syndrome

ANC, Antenatal care

ART, Antiretroviral treatment

ELISA, Enzyme-linked immunosorbent assay

FDA, United States Food and Drug Administration

GHTF, Global Harmonization Task Force

HCT, HIV counselling and testing

HIV, Human immunodeficiency virus

HIVST, HIV self-testing

HTS, HIV testing services

IVDs, *In vitro* diagnostic (medical devices)

M&E, Monitoring and evaluation

NDoH, National Department of Health

PEP, Post-exposure prophylaxis

PMTCT, Prevention of mother-to-child transmission

QA, Quality assurance

QC, Quality control

RDT, Rapid diagnostic test

SAHPRA, South African Health Products Regulatory Authority

SANAC, South African National AIDS Council

STI, Sexually transmitted infection

TB, Tuberculosis

TPP, Target product profile

UNAIDS, Joint United Nations Programme on HIV/AIDS

VCT, Voluntary counselling and testing

WHO, World Health Organization

WHOPQ, WHO Prequalification

## Appendix 3

**TABLE 1-A3:** Attendees: HIV self-testing guidance development group meeting, 17 November 2016, Johannesburg, South Africa.

Attendance	Name	Affiliation
Present	Francois Venter (Co-chair)	Wits Reproductive Health and HIV Institute
	Mohammed Majam (Co-chair)	Wits Reproductive Health and HIV Institute
	Lauren Jankelowitz (Organiser)	Southern African HIV Clinicians Society
	Siraaj Adams	Southern African HIV Clinicians Society
	Pooja Balani	Southern African HIV Clinicians Society
	Sergio Carmona	National Health Laboratory Service
	Thato Chidarikire	National Department of Health
	Francesca Conradie	Southern African HIV Clinicians Society
	Walter Devillé	Ndlovu Care Group/Utrecht University Medical Center
	Krista Dong	iTEACH/Mass General
	Victoria Kazangarare	PSI/Society for Family Health
	Michelle Moorhouse	Southern African HIV Clinicians Society/Wits Reproductive Health and HIV Institute
	Busisiwe Radebe Msimanga	World Health Organization
	Zwoitwaho Nevhutalu	South African National AIDS Council
	Adrian Puren	National Institute for Communicable Diseases
	Naleni Rhagnath (observer)	University of the Witwatersrand
	Hugo Templeman	Ndlovu Care Group
	Renee van der Wiel (observer)	University of the Witwatersrand
Apologies	David Allen	Bill and Melinda Gates Foundation
	Andy Gray	University of KwaZulu-Natal
	Amir Shroufi	Médecins Sans Frontières
	Wendy Stevens	National Health Laboratory Service/University of the Witwatersrand
	Tim Tucker	Strategic Evaluation, Advisory and Development Consulting (SEAD)
	Gilles van Cutsem	Médecins Sans Frontières/University of Cape Town
